# Mechanism of YuPingFeng in the Treatment of COPD Based on Network Pharmacology

**DOI:** 10.1155/2020/1630102

**Published:** 2020-05-27

**Authors:** Yunhong Yin, Jianyu Liu, Mengyu Zhang, Rui Li, Xiao Liu, Yican Yang, Yi-Qing Qu

**Affiliations:** Department of Respiratory and Critical Care Medicine, Qilu Hospital of Shandong University, Jinan 250012, China

## Abstract

YuPingFeng (YPF) granules are a classic herbal formula extensively used in clinical practice in China for the treatment of COPD. However, the pathological mechanisms of YPF in COPD remain undefined. In the present research, a network pharmacology-based strategy was implemented to elucidate the underlying multicomponent, multitarget, and multipathway modes of action of YPF against COPD. First, we identified putative YPF targets based on TCMSP databases and constructed a network containing interactions between putative YPF targets and known therapeutic targets of COPD. Next, two topological parameters, “degree” and “closeness,” were calculated to identify target genes in the network. The major hubs were imported to the MetaCore database for pathway enrichment analysis. In total, 23 YPF active ingredients and 83 target genes associated with COPD were identified. Through protein interaction network analysis, 26 genes were identified as major hubs due to their topological importance. GO and KEGG enrichment analysis results revealed YPF to be mainly associated with the response to glucocorticoids and steroid hormones, with apoptotic and HIF-1 signalling pathways being dominant and correlative pathways. The promising utility of YPF in the treatment of COPD has been demonstrated by a network pharmacology approach.

## 1. Introduction

Chronic obstructive pulmonary disease (COPD) is currently the fourth leading cause of death in the world [1], which will increase to the 3rd leading cause of death by 2020 [2]. Recurrent acute exacerbations of COPD have an even greater impact on patients, resulting in a faster decline in pulmonary function and exponentially increasing the risk of death [3]. The Global Initiative for Chronic Obstructive Lung Disease (GOLD) has indicated that the main treatment goal for stable COPD is to relieve symptoms and reduce the risk of future exacerbations. Therefore, effective prevention of exacerbations is key for the management of COPD [4].

Due to the different pathogenic mechanisms of COPD, the drugs commonly used in clinical practice are inhaled agents, including corticosteroids, *β*2 receptor agonists, and M receptor blockers [5]. However, the existing therapeutics has failed to achieve the expected effect in the treatment of COPD. Therefore, COPD researchers have focused on finding a safer and more effective treatment plan that can improve patient compliance.

With the advancement of traditional Chinese medicine (TCM) research, many have realised the value of TCM, and a large number of clinical reports have shown the advantages of TCM for the treatment of COPD in recent years. The obvious superiority of TCM for COPD treatment is based on overall and comprehensive therapeutic approaches through multiple targets and pathways. The TCM “YuPingFeng” (YPF), which has been used in China for a thousand years, has good clinical efficacy and safety. Recently, several basic studies have confirmed that YPF granules (mainly composed of Radix Astragali, Radix Saposhnikoviae, and Rhizoma Atractylodis Macrocephalae) can significantly improve specific and nonspecific immune functions [6–9]. Clinical studies have also demonstrated that YPF granules reduce the risk of recurrent respiratory infections by regulating the immune system and inhibiting inflammatory cytokines [10], and Ma et al. [11] conducted a multicentre study to elucidate the effects of YPF granules on reducing the risk of acute exacerbations and improving symptom scores in patients with COPD. There are various advantages of YPF, such as good safety, oral administration, pleasant taste, good compliance, and relatively low cost; thus, YPF should be considered for wider clinical application for the treatment of patients with stable COPD. Accordingly, we believe that YPF is a good choice for COPD treatment.

**Figure 1:**
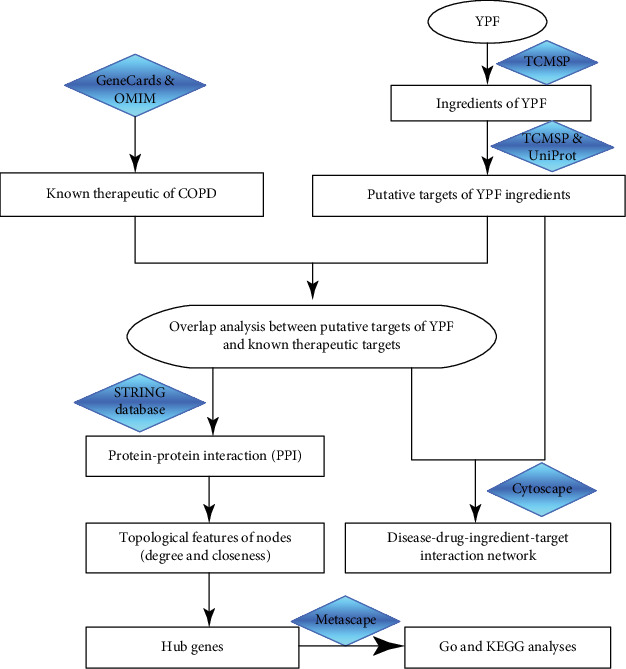
The flowchart of the network pharmacology-based strategy for deciphering the mechanisms of YPF in COPD. Abbreviations: YPF: YuPingFeng; TCMSP: Traditional Chinese Medicine Systems Pharmacology; OMIM: Online Mendelian Inheritance in Man; PPI: protein-protein interaction.

The aim of this research was to explore the mechanism of YPF granules in patients with COPD using network pharmacology, which can predict potential molecules for further analysis. The flowchart of the experimental procedures of our research is shown in Figure 1.

## 2. Materials and Methods

### 2.1. Chemical Ingredient Database Building

To determine the chemical ingredients of the three herbs contained in YPF, we performed a search in the Traditional Chinese Medicine Systems Pharmacology database [12] (TCMSP database, http://tcmspw.com/tcmsp.php, updated on May 31, 2014). TCMSP is a unique systems pharmacology platform of Chinese herbal medicines that captures relationships between drugs, targets, and diseases. The database includes pharmacokinetic properties for natural compounds involving oral bioavailability (OB), drug-likeness (DL), intestinal epithelial permeability, blood-brain barrier, and aqueous solubility, among others. This breakthrough has sparked a new interest in the search for candidate drugs in various types of traditional Chinese herbs [13]. The herbal compounds in orally administered TCM formulae must first overcome the barriers posed by absorption, distribution, metabolism, and excretion (ADME) processes, and only the molecules that pass through those barriers may be active. OB is one of the most important pharmacokinetic parameters in the ADME process. High OB is usually a key indicator for determining the DL index of bioactive molecules. In this study, molecules with an OB value ≥ 33% and DL index ≥ 0.18 are considered to be meaningful active ingredients.

### 2.2. Prediction of Known Therapeutic Targets Acting on COPD

We collected COPD targets from two sources. One was GeneCards (https://www.genecards.org/), a searchable, integrative database that provides comprehensive, user-friendly information on all annotated and predicted human genes. GeneCards automatically integrates gene-centric data from 150 web sources, including genomic, transcriptomic, proteomic, genetic, clinical, and functional information. The keyword “chronic obstructive pulmonary disease” was used to choose a protein-encoded function with a GeneCards Inferred Functionality Score (GIFtS) ≥ 30 as the gene symbol [14]. The GIFtS algorithm uses the wealth of GeneCards annotations to produce scores aimed at predicting the degree of a gene's functionality. Because the degree of known functionality correlates with the amount of research performed on a particular gene or its product, we employ these annotations in a scoring system aimed at inferring functionality. The other resource was the Online Mendelian Inheritance in Man (OMIM) database (http://www.omim.org/, updated August 9, 2019), which catalogues all known diseases with a genetic component, links these diseases to the relevant genes in the human genome, and provides references for further research and tools for genomic analysis of a catalogued gene [15]. We also searched the OMIM database with the query “chronic obstructive pulmonary disease.”

### 2.3. Prediction of Putative Targets of YPF Ingredients

Targets of the active ingredients of all herbs in YPF granules were identified in the TCMSP database. UniProt (http://www.UniProt.org) was used to obtain the official gene symbols of all the targets, and this information was used for subsequent analysis of network pharmacology data.

### 2.4. Screening for Key Targets

The venny R package was employed to map the targets of the three components of YPF granules and known therapeutic targets acting on COPD to construct a Venn diagram. We combined the crossed targets of three drugs and COPD; we deleted duplicate targets and defined these as the key targets for the treatment of COPD.

### 2.5. Network Construction

#### 2.5.1. Disease-Drug-Ingredient-Target Interaction Network

To build the disease-drug-ingredient-target interaction network, we used a practical extraction and reporting (Perl) language, which is an interpreted scripting language. We ran the common drug-disease target, target symbol, and Perl script prepareCyto.pl together to obtain the three drug networks and MoLLISTs, respectively. Then, we used the merge function in Cytoscape software [16] (version 3.6.0, Boston, MA, USA) to construct the disease-drug-ingredient-target interaction network to visualize relationships.

#### 2.5.2. Protein-Protein Interaction Data

Protein-protein interaction (PPI) data were obtained from STRING [17], which covers the majority of known human PPI information. The common target gene was identified with the STRING database online platform (https://string-db.org/) and employed to construct a PPI network model with the species set to “*Homo sapiens*”; the lowest mutual action threshold was set to medium “medium confidence” (>0.4), and other parameters were the default settings.

#### 2.5.3. Identification of Hub Genes

After establishing the PPI network, the Cytoscape plug-in cytoHubba was used to filter out hub genes. An important hub gene can be selected through calculation and analysis of the network structure and the weighted reconnection between nodes. The network visualization software Cytoscape [16] (version 3.6.0, Boston, MA, USA) was adopted to present all of the above networks. This software is very suitable for visualizing networks of molecular interactions and biological pathways. In addition, it provides a powerful set of data integration, analysis, and visualization functions to analyse complex networks. For each node in the interaction network, we selected two indices to calculate topological features. “Degree” is defined as the number of edges to node i; “closeness” is the inverse of the sum of the distance from node i to other nodes. When applying the degree algorithm and closeness algorithm, we considered proteins with ^“^degree^”^ > 20 and ^“^closeness^”^ > 48.8 to be major hubs. Both the hub gene and network were retained, the calculation data were downloaded, the above indicators were sorted, and 26 genes that met the requirements were selected.

#### 2.5.4. GO and KEGG Analyses of Hub Genes

Metascape (http://metascape.org/) is a web-based portal designed to provide a comprehensive gene list annotation and analysis resources for experimental biologists. In terms of design features, Metascape combines functional enrichment, interactome analysis, gene annotation, and membership search to leverage over 40 independent knowledge bases within one integrated portal [18]. Metascape provides a significantly simplified user experience through a one-click Express Analysis interface to generate interpretable outputs. This website is a powerful gene function annotation analysis tool. It is updated once a month to ensure the reliability of the data. Moreover, Metascape integrates data in GO, KEGG, and UniProt, among others. Using multiple authoritative functional databases, we input the 26 genes into the website and ran the Enriched Ontology Clusters program.

## 3. Results

### 3.1. Composite Ingredients and Targets of YPF

According to the two screening conditions of OB value and DL index, a total of 41 chemical ingredients of the three herbal medicines in YPF were retrieved from TCMSP, including 19 ingredients in Radix Astragali (Huangqi), as shown in Table 1, and 15 in Radix Saposhnikoviae (Fangfeng) and 7 in Rhizoma Atractylodis Macrocephalae (Baizhu), as shown in Tables 2 and 3, respectively. Based on the above, we also obtained the three drug targets by TCMSP and the UniProt (http://www.UniProt.org) database to obtain drug targets, which include 94 targets for Huangqi, 26 for Fangfeng, and 10 for Baizhu. In the end, we got a total of 96 targets to remove duplicate values (Additional file 1: Table S1).

### 3.2. Known Therapeutic Targets in COPD

Gene symbols with a protein coding function and a GeneCards Inferred Functionality Score ≥ 30 were selected in GeneCards, which filtered insignificant genes in the database. In total, 3616 known therapeutic targets for COPD were collected from the GeneCards database. In addition, 470 known therapeutic targets for the treatment of COPD were acquired from the OMIM database. After eliminating redundancy, 4037 known therapeutic targets in the treatment of COPD were collected in this study (Additional file 2: Table S2).

### 3.3. Screening for Key Targets

**Figure 2:**
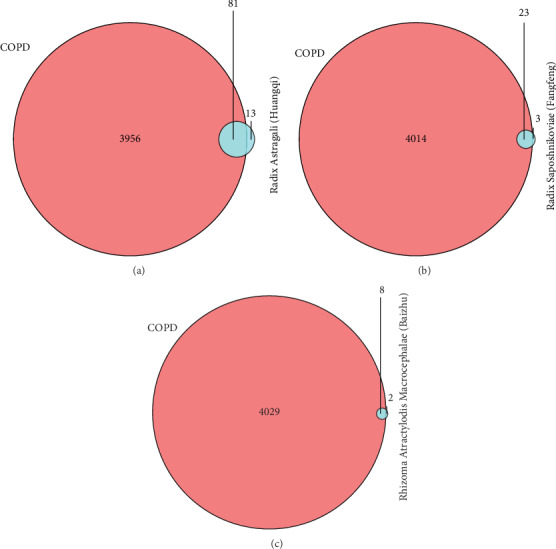
Known therapeutic COPD targets and three herb targets (a) Known therapeutic COPD targets and Radix Astragali (Huangqi) targets. (b) Known therapeutic COPD targets and Radix Saposhnikoviae (Fangfeng) targets. (c) Known therapeutic COPD targets and Rhizoma Atractylodis Macrocephalae (Baizhu) targets.

The 4037 known therapeutic targets for the treatment of COPD were mapped to the three drug targets in YPF using the venny R package to construct a Venn diagram. As shown in Figures 2(a)–2(c), the known therapeutic targets for the treatment of COPD intersecting with Radix Astragali (Huangqi), Radix Saposhnikoviae (Fangfeng), and Rhizoma Atractylodis Macrocephalae (Baizhu) included 81, 8, and 23 targets, respectively. Duplicate targets were deleted, revealing 83 common target genes.

### 3.4. Disease-Drug-Ingredient-Target Interaction Network

**Figure 3:**
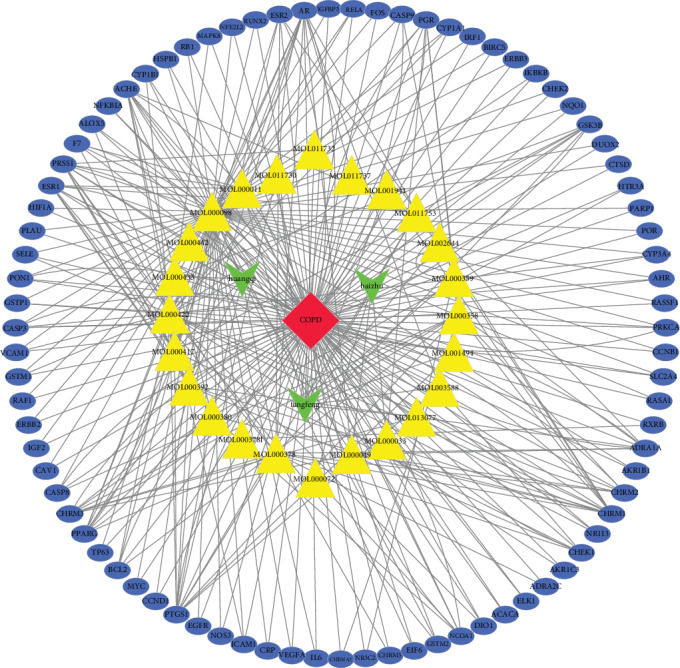
Disease-drug-ingredient-target interaction network. The red diamond node represents the disease COPD, the green v nodes represent the three drugs (Huangqi, Fangfeng, and Baizhu), the yellow triangle nodes represent the 23 active ingredients, the blue ellipse nodes represent the 83 potential target genes, and the lines represent the interactions between them.

We ran the common drug-disease target, target symbol, and Perl script prepareCyto.pl together to obtain drug networks. Then, we used the merge function in Cytoscape software to construct the disease-drug-ingredient-target interaction network, including 1 disease (COPD), three drugs (Huangqi, Fangfeng, and Baizhu), 23 active ingredients, and 83 target genes, as illustrated in the network shown in Figure 3, where the red diamond node represents the disease COPD, the green v nodes represent the drugs, the yellow triangle nodes represent the active ingredients, the blue ellipse nodes represent the potential target genes, and the lines represent the interactions between them. For detailed information about this network, see Additional file 3: Table S3. After the interaction network had been constructed, we got 23 active ingredients, which was different from the total of 41 chemical ingredients obtained at the beginning. The reason is that the target genes of some drug ingredients do not overlap with disease-related genes, which means that these ingredients have no effect on COPD, so they are not shown in the final network.

### 3.5. Construction of the PPI Network

**Figure 4:**
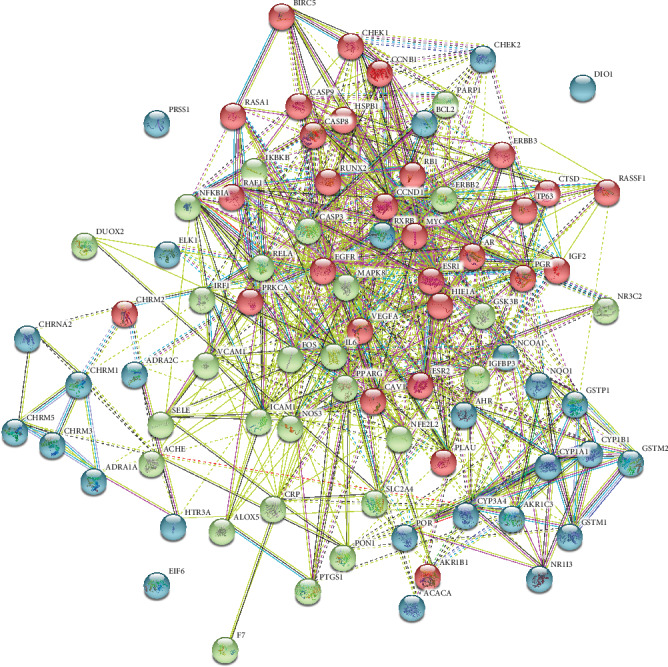
Common-target PPI network: *k*-means clustering was 3, containing 83 nodes, 729 edges, and an average node degree of 17.6.

As depicted in Figure 4, the PPI network of the above-mentioned intersection targets was constructed by the STRING database, with 83 nodes, 729 edges, and an average node degree of 17.6. We used *k*-means clustering (the network was clustered into a specified number of clusters) to obtain 3 clusters.

### 3.6. Identification of Hub Genes

**Figure 5:**
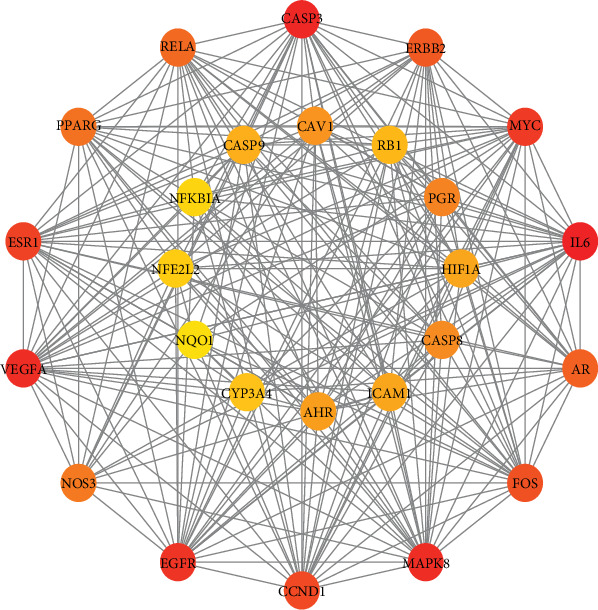
Hub gene interactions in the network: 26 major hubs, according to the degree value, from large to small, the colour gradually changes from red to yellow.

We employed the Cytoscape plug-in cytoHubba to filter the hub genes based on the PPI. We considered proteins with ^“^degree^”^ > 20 and ^“^closeness^”^ > 48.3 to be major hubs. Eventually, 26 major hubs were selected for further study, as shown in the network in Figure 5 and Table 4.

### 3.7. GO and KEGG Analyses of Hub Genes

**Figure 6:**
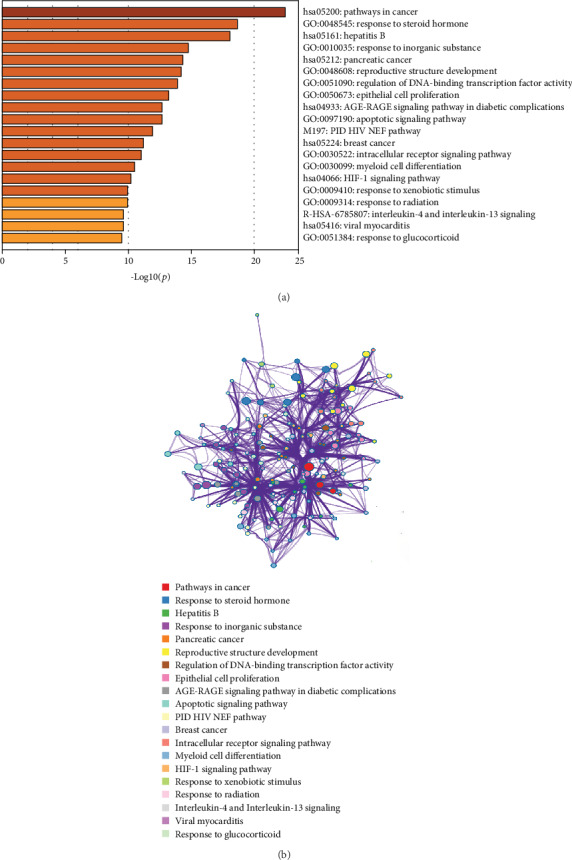
GO and KEGG analyses of the 26 hub genes. (a) Bar graph of the enriched terms across input gene lists coloured according to *p* values: terms with a *p* value < 0.01, a minimum count of 3, and an enrichment factor > 1.5. (b) Network of enriched terms coloured by cluster ID, where nodes that share the same cluster ID are typically close to each other.

Metascape was used for GO and KEGG analyses of the 26 hub genes to obtain enriched ontology clusters, which are expressed in the following two forms: a bar graph, as shown in Figure 6(a), and a coloured cluster ID network, as shown in Figure 6(b). Because this database is a collection of authoritative databases, additional enrichment results combined with COPD were obtained. The functional enrichment mainly included the response to glucocorticoids and steroid hormones, apoptosis signalling pathways, and some inflammation-mediated pathways, such as the AGE-RAGE signalling pathway in diabetic complications and hepatitis B. The HIF-1 signalling pathway was also important. The top 20 clusters with their representative enriched terms are specified in Table 5.

## 4. Discussion

In this study, using the TCMSP analysis platform, 41 active ingredients and 96 drug targets were identified in YPF granules by screening oral bioavailability (OB) and drug-likeness (DL). Next, we constructed a quaternary structure network that contained disease-drug-ingredient targets. According to the results, the most active ingredients and drug targets in YPF granules are Radix Astragali (Huangqi), followed by Radix Saposhnikoviae (Fangfeng) and Atractylodis Macrocephalae Rhizoma (Baizhu). Therefore, the significance of Radix Astragali (Huangqi) in the whole prescription may be the focus of future compatibility studies. After identifying the intersection of the targets of the drugs and disease, 83 common targets were obtained. To narrow the scope, a topology analysis method was adopted, and the degree and closeness were used as the main references based on the established PPI network. Twenty-six hub genes were analysed with the Metascape database to identify enriched GO and KEGG signalling pathways. The main functions of these genes are involved in the response to glucocorticoids and steroid hormones, the apoptotic signalling pathway, and the HIF-1 signalling pathway.

COPD is a global health problem characterized by incomplete reversible airflow limitations and aggravation. Airflow limitation is closely related to structural damage, which causes airway remodelling characterized by chronic inflammation and structural destruction of the airway wall and lung parenchyma. Changes in the composition of the bronchial wall cause the airway space to become constricted and distorted, resulting in the development of emphysema, which is enhanced in airflow resistance, and chronic airflow obstruction. Cytokines and inflammatory mediators play important roles in the pathophysiological processes of COPD [19], and more than 50 types of cytokines are involved in the pathogenesis of COPD [20–22].

We identified hub genes on the basis of PPI network construction, and the top four targets with the highest degrees of freedom were interleukin-6, caspase-3, vascular endothelial growth factor A, and mitogen-activated protein kinase 8. These target genes play important roles in the development of COPD. IL-6 is an important marker of the inflammatory response. Ghobadi et al. [23] found a higher level of IL-6 in the serum with a more severe COPD condition. These results indicate that IL-6 has an important role in the development of COPD. VEGF plays an important role in the repair that occurs after the initiation of airway inflammation, involving a family of ligands (A–D) that bind to three sets of receptors (VEGF-R1, VEGF-R2, and VEGF-R3) [24]. VEGF-A stimulates the endothelial cell and type II cell growth and survival by binding to VEGF receptor 2. Disruption of VEGF signals results in emphysema in adults. More importantly, increasing evidence suggests that human emphysema is associated with decreased VEGF gene expression [25, 26]. VEGF is required for lung maintenance and plays a role in emphysema, which has allowed for the identification of important signalling processes, particularly those involved in oxidative stress and apoptosis. Mitogen-activated protein kinase 8 (MAPK8), also known as JNK1/SAPK1, is an important signalling molecule in the MAPK signal transduction pathway and has various functions, such as in cell proliferation, cell differentiation, and apoptosis [27, 28]. Many scholars believe that airway epithelial cell apoptosis plays an important role in the pathogenesis of COPD and is key in its development [29, 30]. Moreover, related studies have shown that endoplasmic reticulum stress (ERS) may be a very important pathway that mediates apoptosis in airway structural cells [31, 32] and that activation of the endoplasmic reticulum-specific caspase pathway is an important apoptotic pathway [33]. Caspase, a key mediator of programmed cell death (apoptosis), is a frequently activated death protease [34]. The above target genes have key functions in the occurrence and development of COPD and are factors that initiate inflammatory reactions. Regarding functional enrichment granules, the results of our study mainly indicate that YPF granules are associated with the response to glucocorticoids and steroid hormones. Glucocorticoids have potent anti-inflammatory effects and are widely used to treat chronic inflammatory airway diseases [35]. Glucocorticoids bind to the cytoplasmic glucocorticoid receptor (GR), leading to structural changes in the receptor. The complex enters the nucleus of a target cell and binds to DNA, altering the rate of transcription and causing induction or repression of specific genes. Glucocorticoids directly inhibit expression of inflammatory genes and indirectly antagonize transcription factors that promote the transcription of inflammatory genes to exert anti-inflammatory effects [36]. Airway inflammation in COPD is a very complex process, and exploring the specific mechanisms and signalling pathways involved in airway inflammation and finding new therapeutic targets will help to provide new treatment options for this disease.

Imbalanced oxidation/antioxidant function is also an important factor in COPD pathogenesis [37]. Cells and the organism in general are extremely sensitive to hypoxia. Cells specifically regulate expression of certain genes or proteins through oxygen receptors and signal transduction pathways, thus forming a complex oxidative stress response system to maintain the stability of the internal environment [38]. HIF-1 is the only transcription factor that has been found to be specifically active under hypoxic conditions and plays a nonnegligible role in hypoxia regulation. In recent years, studies showing that HIF-1 mediates gene transcription and regulates hypoxia have received increasing attention. HIF-1 is widely involved in multiple cellular signalling pathways and is a transduction hub that mediates hypoxia signalling [39]. In addition, crossregulation exists between the HIF-1 pathway and other signalling pathways, thus forming the specific yet diverse HIF-1-mediated cellular hypoxia response pathway [40]. HIF-1 is composed of an oxygen-sensitive HIF-1*α* subunit and a constitutively expressed cytoplasmic HIF-1*β* subunit. HIF-1*α* is a major regulator of oxygen homeostasis and regulates the pathophysiology of hypoxia [41]. The main mechanism is that hypoxia enhances the stability of hypoxia-inducible factor 1-alpha (HIF-1*α*) and promotes the binding of HIF-1 to HRE, thereby inducing activation of hypoxia-sensitive target genes and further promoting the cellular response to hypoxia. This study found that YPF granules play a role in COPD treatment through the HIF-1 signalling pathway. Nonetheless, the specific mechanism of action of the pathway has not yet been elucidated, which should be addressed in subsequent studies.

In summary, the potential biological mechanism of YPF granules in the treatment of chronic obstructive pulmonary disease involves multiple components, targets, and channels, and the therapeutic effects occur through pathways involving glucocorticoids and steroid hormones. The pharmacodynamic mechanism of YPF granules in COPD, including the inflammatory mechanism, apoptosis, and HIF-1 signalling pathway, was elucidated from a holistic and systematic perspective. This study provides a scientific basis for the subsequent development and utilization of YPF granules. Of course, because only network pharmacology techniques were applied to examine the active components and targets of YPF granules, there may be experimental limitations such as inconsistent original information, limited information on drug targets and small molecule compounds, and biased information in the database due to the popularity of some topics. Therefore, experimental research is needed to verify the results of this study, especially the mechanism through which YPF granules and its components regulate apoptosis and the HIF-1 signalling pathway.

## 5. Conclusion

The mechanism of action of YPF in COPD involves multiple compounds, targets, and pathways. The therapeutic effects of YPF in COPD may be dependent on the response to glucocorticoid and steroid hormones and pathways related to HIF-1 signalling and apoptotic signalling. The systems pharmacology approaches developed in our study provide an alternative strategy for comprehensively understanding the mechanisms of YPF in COPD.

## Figures and Tables

**Table 1:** Ingredients of Huangqi.

Mol ID	Molecule name	OB (%)	DL
MOL000211	Mairin	55.38	0.78
MOL000239	Jaranol	50.83	0.29
MOL000296	Hederagenin	36.91	0.75
MOL000033	(3S,8S,9S,10R,13R,14S,17R)-10,13-Dimethyl-17-[(2R,5S)-5-propan-2-yloctan-2-yl]-2,3,4,7,8,9,11,12,14,15,16,17-dodecahydro-1H-cyclopenta[a]phenanthren-3-ol	36.23	0.78
MOL000354	Isorhamnetin	49.6	0.31
MOL000371	3,9-Di-O-methylnissolin	53.74	0.48
MOL000374	5′-Hydroxyiso-muronulatol-2′,5′-di-O-glucoside	41.72	0.69
MOL000378	7-O-Methylisomucronulatol	74.69	0.3
MOL000379	9,10-Dimethoxypterocarpan-3-O-*β*-D-glucoside	36.74	0.92
MOL000380	(6aR,11aR)-9,10-Dimethoxy-6a,11a-dihydro-6H-benzofurano[3,2-c]chromen-3-ol	64.26	0.42
MOL000392	Formononetin	69.67	0.21
MOL000398	Isoflavanone	109.99	0.3
MOL000417	Calycosin	47.75	0.24
MOL000422	Kaempferol	41.88	0.24
MOL000433	FA	68.96	0.71
MOL000438	(3R)-3-(2-Hydroxy-3,4-dimethoxyphenyl)chroman-7-ol	67.67	0.26
MOL000439	Isomucronulatol-7,2′-di-O-glucosiole	49.28	0.62
MOL000442	1,7-Dihydroxy-3,9-dimethoxy pterocarpene	39.05	0.48
MOL000098	Quercetin	46.43	0.28

**Table 2:** Ingredients of Fangfeng.

Mol ID	Molecule name	OB (%)	DL
MOL000011	(2R,3R)-3-(4-Hydroxy-3-methoxy-phenyl)-5-methoxy-2-methylol-2,3-dihydropyrano[5,6-h][1,4]benzodioxin-9-one	68.83	0.66
MOL011730	11-Hydroxy-sec-o-beta-d-glucosylhamaudol_qt	50.24	0.27
MOL011732	Anomalin	59.65	0.66
MOL011737	Divaricatacid	87	0.32
MOL001941	Ammidin	34.55	0.22
MOL011749	Phelloptorin	43.39	0.28
MOL011753	5-O-Methylvisamminol	37.99	0.25
MOL002644	Phellopterin	40.19	0.28
MOL000359	Sitosterol	36.91	0.75
MOL000358	beta-Sitosterol	36.91	0.75
MOL001494	Mandenol	42	0.19
MOL001942	Isoimperatorin	45.46	0.23
MOL003588	Prangenidin	36.31	0.22
MOL007514	Methyl icosa-11,14-dienoate	39.67	0.23
MOL013077	Decursin	39.27	0.38

**Table 3:** Ingredients of Baizhu.

Mol ID	Molecule name	OB (%)	DL
MOL000020	12-Senecioyl-2E,8E,10E-atractylentriol	62.4	0.22
MOL000021	14-Acetyl-12-senecioyl-2E,8E,10E-atractylentriol	60.31	0.31
MOL000022	14-Acetyl-12-senecioyl-2E,8Z,10E-atractylentriol	63.37	0.3
MOL000028	*α*-Amyrin	39.51	0.76
MOL000033	(3S,8S,9S,10R,13R,14S,17R)-10,13-Dimethyl-17-[(2R,5S)-5-propan-2-yloctan-2-yl]-2,3,4,7,8,9,11,12,14,15,16,17-dodecahydro-1H-cyclopenta[a]phenanthren-3-ol	36.23	0.78
MOL000049	3*β*-Acetoxyatractylone	54.07	0.22
MOL000072	8*β*-Ethoxy atractylenolide III	35.95	0.21

**Table 4:** Information on hub genes.

Gene symbol	Gene name	Degree	Closeness
IL-6	Interleukin-6	50	64.33333
CASP3	Caspase-3	48	63.33333
MAPK8	Mitogen-activated protein kinase 8	47	62.5
VEGF-A	Vascular endothelial growth factor A	47	62.5
EGFR	Epidermal growth factor receptor	46	62.16667
MYC	Myc protooncogene protein	45	61.5
ESR1	Estrogen receptor	44	61
CCND1	G1/S-specific cyclin-D1	42	60
FOS	Protooncogene c-Fos	38	58.5
ERBB2	Receptor tyrosine-protein kinase erbB-2	36	56.83333
AR	Androgen receptor	34	56
RELA	Transcription factor p65	32	55.5
PPARG	Peroxisome proliferator activated receptor gamma	32	55
NOS3	Nitric oxide synthase, endothelial	29	53.83333
PGR	Progesterone receptor	28	53
CASP8	Caspase-8	27	52
CAV1	Caveolin-1	26	51.5
AHR	Aryl hydrocarbon receptor	25	51.16667
ICAM1	Intercellular adhesion molecule 1	24	51
HIF-1*α*	Hypoxia-inducible factor 1-alpha	24	51
CASP9	Caspase-9	24	50.66667
RB1	Retinoblastoma-associated protein	22	49.66667
CYP3A4	Cytochrome P450 3A4	21	49.5
NFE2L2	Nuclear factor erythroid 2-related factor 2	21	49.33333
NFKBIA	NF-kappa-B inhibitor alpha	20	48.66667
NQO1	NAD(P)H dehydrogenase [quinone] 1	20	48.33333

**Table 5:** Top 20 clusters with representative enriched terms (one per cluster).

GO	Category	Description	Count	%	Log10(*p*)	Log10(*q*)
hsa05200	KEGG pathway	Pathways in cancer	17	65.38	-24.08	-19.76
GO:0048545	GO biological processes	Response to steroid hormone	14	53.85	-18.36	-14.34
GO:0010035	GO biological processes	Response to inorganic substance	15	57.69	-17.79	-14.07
hsa05161	KEGG pathway	Hepatitis B	11	42.31	-17.79	-14.07
hsa05212	KEGG pathway	Pancreatic cancer	8	30.77	-14.63	-11.16
GO:0051090	GO biological processes	Regulation of DNA binding transcription factor activity	12	46.15	-14.15	-10.84
GO:0048608	GO biological processes	Reproductive structure development	12	46.15	-14.15	-10.84
GO:0050673	GO biological processes	Epithelial cell proliferation	12	46.15	-13.98	-10.75
hsa04933	KEGG pathway	AGE-RAGE signalling pathway in diabetic complications	8	30.77	-13.06	-9.94
GO:0097190	GO biological processes	Apoptotic signalling pathway	12	46.15	-12.44	-9.45
M197	Canonical pathways	PID HIV NEF Pathway	6	23.08	-11.88	-8.97
hsa05224	KEGG pathway	Breast cancer	8	30.77	-11.73	-8.87
GO:0060749	GO biological processes	Mammary gland alveolus development	5	19.23	-11.09	-8.36
GO:0030099	GO biological processes	Myeloid cell differentiation	10	38.46	-11.05	-8.34
hsa04066	KEGG pathway	HIF-1 signalling pathway	7	26.92	-10.95	-8.27
GO:0009410	GO biological processes	Response to xenobiotic stimulus	9	34.62	-10.9	-8.22
R-HSA-6785807	Reactome gene sets	Interleukin-4 and Interleukin-13 signalling	7	26.92	-10.75	-8.1
GO:0009314	GO biological processes	Response to radiation	10	38.46	-10.74	-8.1
hsa05416	KEGG pathway	Viral myocarditis	6	23.08	-10.44	-7.85
GO:0051384	GO biological processes	Response to glucocorticoid	7	26.92	-9.82	-7.34

“Count” is the number of genes in the user-provided lists that are associated with the given ontology term. “%” is the percentage of all of the user-provided genes that are found in the given ontology term (only input genes with at least one ontology term annotation are included in the calculation). “Log10(*p*)” is the *p* value in log base 10. “Log10(*q*)” is the multitest-adjusted *p* value in log base 10.

## Data Availability

Not applicable.

## Supplementary Materials

Supplementary Materials Table S1: putative targets of each herb contained in YPF. Table S2: known therapeutic targets for COPD. Table S3: disease-drug-ingredient-target interaction network.
